# PD44 - In vitro fertilisation is positively associated with prevalence of asthma in childhood

**DOI:** 10.1186/2045-7022-4-S1-P44

**Published:** 2014-02-28

**Authors:** George V Guibas, George Moschonis, Paraskevi Xepapadaki, Eirini Roumpedaki, Odysseas Androutsos, Yannis Manios, Nikolaos G Papadopoulos

**Affiliations:** 1 Allergy Department, 2nd Pediatric Clinic, University of Athens, Athens, Greece; 2 Department of Nutrition and Dietetics, Harokopio University, Athens, Greece

## Background

Research on potential perinatal risk factors for asthma, has recently attracted considerable attention. Asthma could be associated with *In vitro* fertilization (IVF) *via* epigenetic modification of DNA by IVF drugs/hormones or *via* a genetic link of asthma with parental subfertility. Nevertheless, evidence of an asthma/IVF correlation is scarce and inconclusive. We therefore opted to explore a potential link, in a cross-sectional population-based study in preadolescent children.

## Methods

Wheeze in the last 12 months (current), wheeze ever, physician-diagnosed asthma and method of conception were recorded from questionnaires filled in by the parents of 2016 Greek children aged 9-13. Perinatal data was collected from their medical records and the questionnaires; anthropometric measurements were conducted. Logistic regression models were build in the Statistical Package for Social Sciences (SPSS version 20.0), with the wheeze/asthma variables as main outcomes. A two-tailed p value less that 0.05, was considered statistically significant.

## Results

IVF correlated with physician-diagnosed asthma (OR=2.69, 95%CI=1.5-4.79, p=0.001) and ever wheeze (OR=2.01, 95%CI=1.07-3.78, p=0.03) but not with current wheeze (p>0.05) in univariate unadjusted regression models. The link of IVF with asthma remained significant (OR=2.04, 95%CI=1-4.15, p=0.05) after adjustment for a wide array of potential confounding factors (maternal prenatal smoking, gestational age, single or multiple gestation, method of delivery, birth weight, gender, parity, breastfeeding, parental educational level, passive smoking at home, current BMI, family status, mother occupation, people involved in daily childcare and municipality/county/urbanity wherein the subjects resided). However the link with ever wheeze was lost (p>0.05).

## Conclusions

Conception via *in vitro* fertilization may predispose children to future asthma development.

